# Scan for Motifs: a webserver for the analysis of post-transcriptional regulatory elements in the 3′ untranslated regions (3′ UTRs) of mRNAs

**DOI:** 10.1186/1471-2105-15-174

**Published:** 2014-06-08

**Authors:** Ambarish Biswas, Chris M Brown

**Affiliations:** 1 Department of Biochemistry, Genetics Otago, University of Otago, Dunedin, New Zealand; 2 Genetics Otago, University of Otago, Dunedin, New Zealand

**Keywords:** Untranslated region, microRNA, RNA binding protein, Translational control

## Abstract

**Background:**

Gene expression in vertebrate cells may be controlled post-transcriptionally through regulatory elements in mRNAs. These are usually located in the untranslated regions (UTRs) of mRNA sequences, particularly the 3′UTRs.

**Results:**

Scan for Motifs (SFM) simplifies the process of identifying a wide range of regulatory elements on alignments of vertebrate 3′UTRs. SFM includes identification of both RNA Binding Protein (RBP) sites and targets of miRNAs. In addition to searching pre-computed alignments, the tool provides users the flexibility to search their own sequences or alignments. The regulatory elements may be filtered by expected value cutoffs and are cross-referenced back to their respective sources and literature. The output is an interactive graphical representation, highlighting potential regulatory elements and overlaps between them. The output also provides simple statistics and links to related resources for complementary analyses. The overall process is intuitive and fast. As SFM is a free web-application, the user does not need to install any software or databases.

**Conclusions:**

Visualisation of the binding sites of different classes of effectors that bind to 3′UTRs will facilitate the study of regulatory elements in 3′ UTRs.

## Background

The untranslated regions of mRNA sequences (UTRs) include most of the experimentally determined regulatory elements (REs) [1,2]. This post-transcriptional regulatory information can affect the site at which a mRNA is polyadenylated, and then how, when and where it is translated [3,4]. A number of tools and methods have been developed to identify *cis*-regulatory elements (CREs), many focusing on individual types of CREs in single sequences [5,6]. These may ignore the detection of other types of CREs in the neighboring regions [7,8]. For example, although there are a large number of algorithms to predict microRNA (miRNA) binding sites, reviewed in [9,10], only one has included specific consideration of a nearby RNA binding protein (RBP) site [11]. However, some miRNA targets are known to be affected by the presence of other elements or sequences nearby [1,11-13]. Most regulatory elements are quite small (<12 bases) and many *in silico* predictions have high false positive rates. Visualisation of potential sites could improve the utility of predictions.

Some complex RNA elements can be both miRNA target sites and be bound by proteins [3,14,15]. Recent publications have shown evidence that specific types of miRNAs and RBPs work in concert to influence transcript decay [11,16,17] or translation [13] and this synergy has been included in some computational analyses for proteins [18] and miRNAs [19].

In many studies one specific gene of interest from a single species is being analysed. Recently developed systems: RegRNA 2.0 [2], AURA [20], ARESite [6], and UTRdb [21] have provided increasing support for this type of analysis. However, the analysis of sequence alignments, a representation of overlapping identified elements, E-value cutoff, and the ability to include custom sequence motifs in the analysis, are not currently available in a single tool. Scan for Motifs provides this for 3′UTR regions. It is primarily aimed at the analysis of human 3′UTRs, but can be used for any species sequences, alignments, or any part of the mRNA.

## Implementation

**Figure 1:**
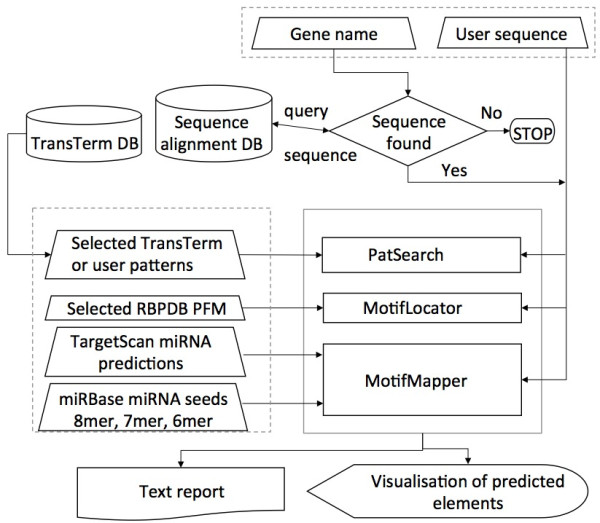
**Outline of the main modules and steps involved in a Scan for Motifs analysis.** The user input sections are in dashed boxes. User selected analyses are executed on demand. TargetScan predictions are also re-mapped to the genomic alignments using PERL scripts (labelled MotifMapper).

The analysis has three phases: 1. accepting user input, 2. analysing the sequence(s), and 3. interactive visualization of the results (Figure 1). The processes to identify and visualise the regulatory elements for any selected gene or given sequence(s) is done in parallel for speed. Input can be the name of a human gene (e.g. TNF) in which case the standard TargetScan/UCSC vertebrate alignment will be used. However, the user can also input any sequence or alignment. The server is a pure LAMP (Linux, Apache, MySQL and Perl) implementation providing speed and stability, using HTML, JavaScript and AJAX to provide seamless user interaction throughout the analysis. SFM has been tested on commonly used web-browsers: Chrome, Firefox, Safari and Explorer 10 or later.

### Data analysed

The RNA-Binding Protein DataBase (RBPDB) contains a collection of experimentally verified RNA binding sites, manually curated from literature. It currently contains binding data on 272 RBPs, but only 69 that have motifs in position frequency matrix (PFM) format most useful for SFM analysis. These PFM can be used to distinguish between good and poor matches for short motifs. The other individual binding site sequences from RBPDB could also be user specified (e.g. CAUY). Other user specified sequences, regular expressions, or matrices can also be used in PatSearch format [22].

Published miRNA sequences are from miRBase [23]. The mature miRNA sequences were downloaded from miRBase website (file:mature.fa), processed (reverse complemented and 8 leading seed bases extracted) to get a list of 2042 named 8mer seeds and stored in a reference text file. The 6mer seed is the middle 6-bases, and both the two overlapping 7mers are used (7mer-A1, denoted A1 in the output, and 7mer-M8) [8].

The 3′UTR alignments used were obtained from TargetScan (v.6.2) along with the microRNA-binding site related files (miR Family, Predicted Conserved Targets Info, Conserved Family Info) [8]. The ‘UTR_Sequences’ file holds multiple sequence alignments (MSA) of 23 vertebrate genomes aligned to human, extracted from the USCC human genome (hg18) databases by the TargetScan authors. The human specific sequences were extracted and the positional information for the miR-binding sites provided in “Predicted Conserved Targets Info” file was compared to and updated where needed) against the latest release of hg19 database (from UCSC). A bed format MySQL database table was created to hold the positional information for each of these miR-binding sites.

A custom Perl script was written and used for checking and updating the positional information as above. The program uses sequence similarity between the latest release of hg19 (from UCSC) and the UTR sequences from the TargetScan website. In most of the cases the sequences were 100% identical. For 27 genes the sequences were found to be different in length, the TargetScan prediction data for these were discarded, as they could not be unambiguously assigned to the sequence.

### Accepting user input

**Figure 2:**
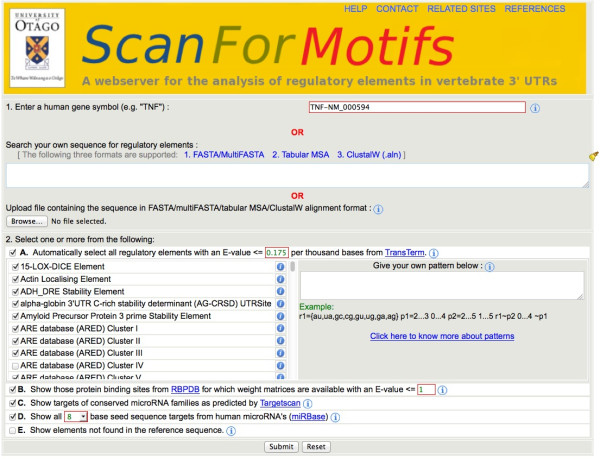
**The input section of Scan for motifs showing the range of supported regulatory elements and background controls.** For a pre-aligned human 3′ UTR (e.g. TNF-NM_000594) it defaults to searching for over 60 TransTerm regulatory elements with expectations of E-value ≤ 0.175 by chance in typical human 3′ UTR (~1000 nt) (A in Figure) and TargetScan miRNA binding site predictions for ~150 conserved miRNA families (C). In this case the sites for RNA binding proteins with E-values ≤ 1.0 per thousand (B) and miRBase 8mer seeds (D) are also selected.

The user input is of two types, i) query sequence(s) and ii) query element(s). Figure 2 shows the different input options available in SFM web-server.i) Query sequence. Option 1 in Figure 2 shows the different types of sequence that is accepted by SFM. It supports input of a standard human gene symbol (i.e. LIN28A) given as source of the query sequence. In such cases relative sequence alignments of 23 vertebrates (including human) will be retrieved from previously processed sequences using the inputted gene symbol and used as query sequence. Alternately, users can input FASTA/multiFASTA/clustalW alignments as well as tabular multiple sequence alignment (MSA) formatted sequences as query sequence. SFM supports assigning reference sequence when the query sequence has more than one sequence. If a human gene symbol was used to get the input sequence, the reference sequence is assigned to be human. In all other cases, the first sequence is considered to be the reference sequence.ii) Query elements. Option 2. A-E in Figure 2 shows the range of query elements expect value controls available in SFM. All the 77 Transterm elements (option 2. A in Figure 2) are associated with an background Expect-value (E-value) frequency of occurrence per thousand bases. These E-values were calculated by first creating a background set by dinucleotide shuffling a non-redundant set of 18,895 human 3′UTR sequences, then searching these with each of the elements. For example an expect value of 0.175 (the default) corresponds to an expectation that each element may appear on average by chance 0.175 times in a typical analysis of one human 3′ UTR of 1000 nt. Elements can be automatically selected/deselected by changing the E-value cutoff (shown in the red box in option 2. A in Figure 2.2). Additionally, users can give their own pattern or sequence motif (e.g. AUAGGGU), which will be searched along with the other selected elements against the query sequence(s) using PatSearch.Similarly, option 2.B-D (Figure 2) shows the elements from RBPDB, TargetScan and miRBase respectively along with the options to limit the hits based on MotifLocator calculated matches using the 69 RBPDB PFM. The TargetScan elements are available only when a published human gene symbol is used.Option 2.E (Figure 2) The default behaviour is only to show elements in non-reference sequences if also found in the reference sequence (e.g. human). This can be disabled using this option.

### Processing sequences

Upon receiving the input, SFM searches for the query elements using independent parallel processes, where the output from one process is not affected by another process (Figure 1). Irrespective of the input sequence types, all sequences are converted to FASTA format. The patterns from the selected TransTerm elements and user given pattern(s) are used to search the input sequences using PatSearch [22]. The 69 RNA binding protein PFM from RBPDB are used to search the sequences with MotifLocator [24]. The TargetScan miRNA binding sites and their position of occurrences were retrieved from the MySQL database table (see section 2.2.1) by using the input human gene symbol and mapped on the query sequences using PERL scripts labelled MotifMapper in Figure 1. Based on the user given seed length (6, 7 or 8 nucleotides), a list of seed sequences are created from the 2042 seed sequences. As one seed sequence can be associated with multiple miRNAs in a family, a non-redundant list of seed sequences was made. These sequences were used to search the query sequence(s) using PERL RegEx (regular expressions). Once all the processes are finished, the results from these processes are combined and sent to the visualisation module.

### Interactive output

The output is shown on a scrollable alignment with links to further information and the ability to show or hide specific components of the complex results.

## Results and discussion

The SFM web-server analyses sequences that may be aligned vertebrate UTRs, or user inputted sequences or alignments (Figure 1). Five types of elements are searched for in these sequences.

(i) Regulatory elements from the TransTerm database, which includes relevant UTRSite and ARED elements. This provides a curated collection of CREs that function as translational control elements in mRNAs. The computational models (elements) are selected by the user, and/or filtered on empirically determined background frequencies in a shuffled control set. Matches are identified using PatSearch [22].

(ii) RBP binding sites represented as position frequency matrices (PFM) from the RBPDB [25]. Matches are identified using MotifLocator [24] with a user specified E-value filter.

(iii) MicroRNA target sites predicted by TargetScan 6.2 [8]. TargetScan was chosen as it is widely used, and predicts sites on vertebrate alignments

(iv) Human miRNAs 6 to 8 base seed sequences [23] using MotifMapper. This simple prediction is intended to allow visualisation of most of the potential miRNA binding sites, including likely false positives, if the user desires to.

(v) User defined patterns in PatSearch format [21]. PatSearch allows searches for simple strings, optionally with mismatches insertions and deletions (e.g. GNGNCC), but also more complex elements (e.g. GCG 3…7 GCG, two GCG separated by 3–7 bases) and RNA secondary structures (e.g. p1 = 10…10 4…7 ~ p1, a ten base stem with a loop of 4–7 bases). A full description of the syntax is presented in the help on the SFM server.

**Figure 3:**
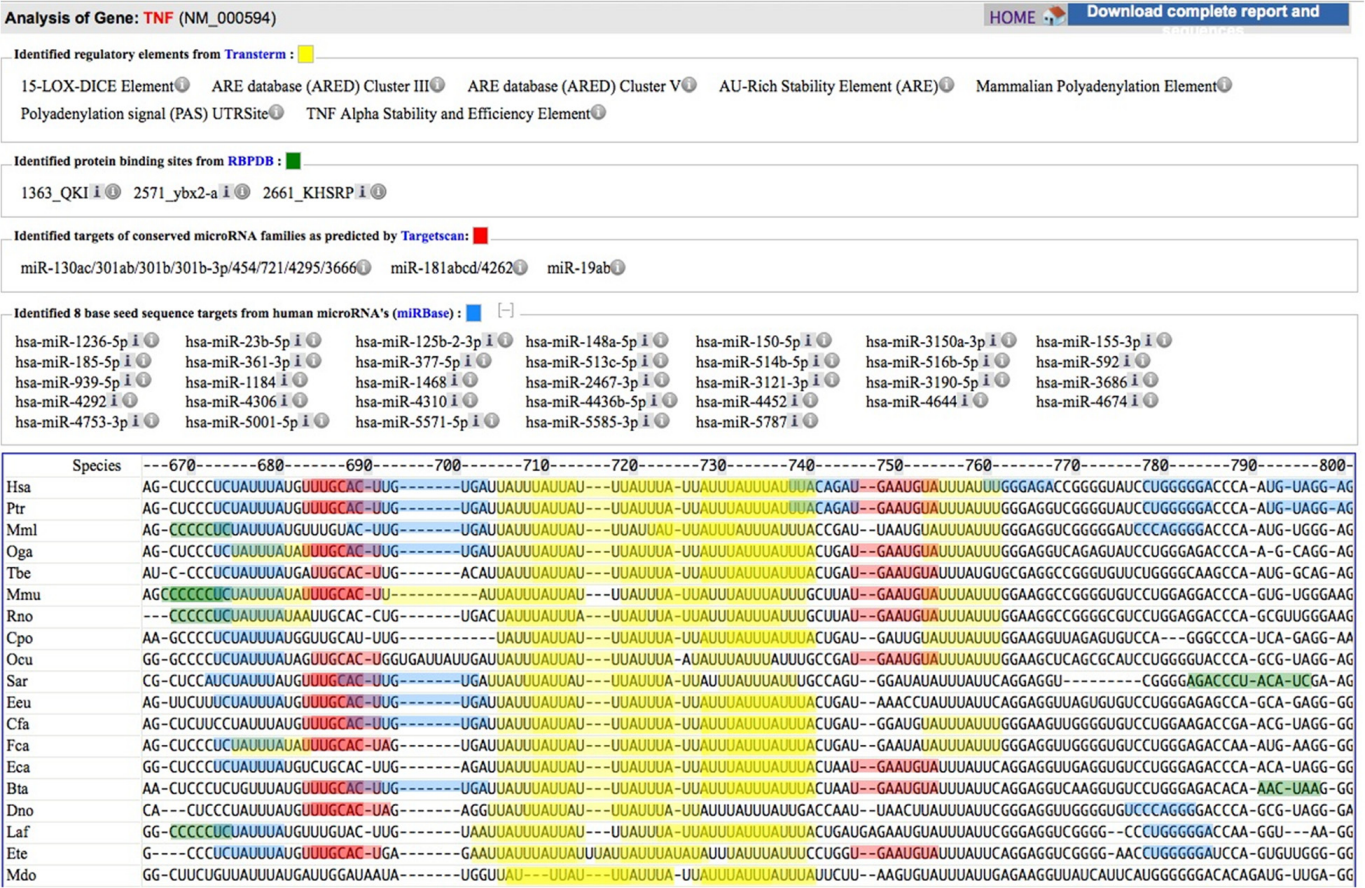
**Scan for motifs’s interactive graphical output for the vertebrate Tumor Necrosis Factor (TNF) 3′UTRs, using the settings shown in Figure**2**.** Known protein and miRNA sites are detected, and additional predictions are made. The experimentally confirmed and conserved ARE mRNA stability elements are shown in the centre (~710-740, yellow). These are flanked by TargetScan miRNA target predictions (red), miR-19a and miR-181a are known to target these sites. The miR-130 TargetScan prediction almost completely overlaps the miR19 site (left, two intensities of red). Some of the additional predictions include patterns of lower specificity (green and blue) are not conserved and may be false positives (e.g. the KHSRP protein binding site to the left (green ~670), or the miR-150-5p (blue ~760). However miR-125 (blue 8mer seed match ~690) does target this UTR. The results can be downloaded for further study (upper right). See the Results and Discussion section for further analysis.

On completion of the individual processes, the results are compiled and presented as interactive visualisation (Figure 3). As an example, we use the well-studied tumor necrosis factor alpha (TNF) 3′ UTR. TNF is a multifunctional cytokine, it regulates the expression of other genes in inflammation and other processes and its expression is regulated at main steps [26]. The TNF 3′ UTR has been shown to be targeted by both proteins and miRNA [13,27] and is a classic example of an ARE containing mRNA. MicroRNAs that are confirmed to target this UTR in mammals are miR-16 [28], miR-19a [29], miR-125b [30], miR-130 [31], miR-181a [32], miR-301 [31]. Unusually, a miR-369-3p containing RNA-protein complex binds to targets within the ARE and activates or represses translation in the cell cycle [13]. This ARE may also be bound by the RNABP tristetraprolin (TTP) to repress translation [33].

In the SFM analysis using the settings in Figure 2, highlights several types of elements from the TransTerm database (Figure 3, yellow): the AU rich element (ARE) is represented by hits from three overlapping descriptions (Background E-value per thousand bases 0.06, 0.12, 0.12 respectively, Figure 3) [34]; TNF Alpha Stability and Efficiency Element (E-value 0.000008) [35]; and two descriptions of a Polyadenylation Element at the 3′ end (E-value 0.03, 0.02). These are all present in a similar position in the alignment across vertebrates, and the 9–12 base core ARE [34] is repeated [34]. The two predicted stability elements in the TNF 3′ UTR have been verified experimentally [27,35], and the polyadenylation signal has a clear match to the consensus (AAUAAA). In addition a 15-LOX-DICE element is predicted (E-value 0.01) in the same location in only 5 of 17 species. From the information linked from the small ‘i’ to the TransTerm entry it can be found that the 15-LOX-DICE is known to have a role in regulating mRNA stability of mRNAs in early erythropoiesis [36]. This may be a false positive, or a novel finding requiring further investigation.

Three predicted overlapping miRNA binding sites are shown (Figure 3, red). Interesting they flank the ARE. Each site links to the family of miRNAs that could bind this seed (e.g. miR181abcd/462) this data is inherited from the TargetScan families and predictions [8]. Included in these predictions are miR-19a, miR-181a, miR-130/miR-301 they have been shown to target these regions in the TNF UTR.

Not predicted with the conservative default SFM parameters are two sites for miR-369-3p within the ARE [13]. These could be shown when 7mer miRBase seeds (miR-369-3p, UAUUAUU) are selected overlapping the ARE. These miR-369-3p sites are also conserved in the alignment. The TargetScan analysis with 153 ‘broadly conserved’ and ‘conserved’ miRNA families did not predict this site, as miR-369 is poorly conserved [8] so they are not shown in the results from this analysis (Figure 3 red). However, TargetScan does not predict this known site at all (TargetScan webserver) possibly due to the weak AU base pairing within this site.

Such short matches (6mer, 7mer) should be interpreted with caution, as there are over 4000 possible 7mer seeds from the 2043 mature human miRNA seeds in miRBase. This resulted in over 200 hits in the 17,000 nt TNF UTR alignment. However, most of these matches are not conserved (not present in a similar locations in the alignment) and can therefore be identified as likely false positives by visual inspection of the SFM output.SFM visually represents different types of element in one display (Figure 3). On the output page it also provides the user the choice to include/exclude any sets of elements in the analysis, as well as only showing elements also found in the reference sequence (e.g. human, when a gene symbol is used as input). Along with the graphical display, SFM also provides a text report listing the entire user input (selections and input sequence) as well as output of each individual search process.

## Conclusions

SFM is a free web-application, allowing researchers to use a single tool to identify and investigate a range of CREs on both alignments and single sequences. Notably, these include both protein binding sites (Transterm, UTRSite, ARED) and miRNA binding sites (TargetScan, miRBase seed match). These elements come from well-documented databases and are cross-referenced to these. We believe that SFM will be particularly useful for researchers to uncover relationships among different classes of post-transcriptional regulatory elements.

## Availability and requirements

**Project name:** Scan for motifs.

**Project home page:**http://bioanalysis.otago.ac.nz/sfm/.

**Operating system:** Platform independent.

**Programming language:** Perl, MySQL.

**Other requirements:** none.

**License:** Free to use.

**Any restrictions to use by non-academics:** None.

## Competing interests

The authors declare that they have no competing interests.

## Authors’ contributions

AB designed and developed the software. CMB conceived of the application, supervised it, and tested it. Both authors wrote, read and approved the final manuscript.
